# Editorial: Brain Networks in Aging: Reorganization and Modulation by Interventions

**DOI:** 10.3389/fnagi.2017.00425

**Published:** 2017-12-19

**Authors:** Junfeng Sun, Chunbo Li

**Affiliations:** ^1^School of Biomedical Engineering, Shanghai Jiao Tong University, Shanghai, China; ^2^Brain Science and Technology Research Center, Shanghai Jiao Tong University, Shanghai, China; ^3^Shanghai Key Laboratory of Psychotic Disorders, Shanghai Mental Health Center, Shanghai Jiao Tong University School of Medicine, Shanghai, China; ^4^Center for Excellence in Brain Science and Intelligence Technology (CEBSIT), Chinese Academy of Sciences, Shanghai, China

**Keywords:** brain network, aging, cognitive training, EEG, MRI

Older adults undertake reduction of cognitive function and brain reorganization in forms of regional brain activity, inter-region connectivity, and brain network topology in both function and structure during aging process. Various theories, such as compensation and dedifferentiation, have been proposed to explain the underlying mechanisms of these aging-related changes. As advanced cognitive function is generally achieved by the integration of multiple brain regions, brain network is used to characterize the integration and organization of brain regions that may be spatially far separated. Furthermore, interventions such as cognitive training and noninvasive neuromodulation have been demonstrated to be promising tools to maintain or even improve the cognitive function of older adults during specific aging states. Focusing on the brain network in aging, this Research Topic mainly collected three set of studies.

The first set of studies include six papers on the cognitive function of normal older subjects and mild cognitive impairment (MCI) patients in behavior and neural activities based on Electroencephalography (EEG). Hong et al. investigated the aging effects on brain networks during either response execution (Go) or response inhibition (NoGo) condition with graph theoretical analysis. Results showed that the functional brain networks of both young and old subjects had prominent but different small-world properties in Go and No-go tasks, and older adults showed stronger task-modulated effects on small-world properties than younger adults (Hong et al.). In another study, Hsieh et al. examined the older adults' inhibition strategies to irrelevant distraction and conflict distraction respectively in Go/NoGo tasks. Three studies investigated aging effects on attention. Kaufman et al. studied the aging effects on three attentional networks (alerting, orienting, and executive control) from both behavior performance and event-related potential (ERP), and showed that older adults had alerting deficits in attention compared with younger adults (Kaufman et al.). In another study based on an oddball task, Kaufman et al. demonstrated that older subjects had enhanced scalp signals during an early stage of visual processing, which correlated with neural activity in primary visual cortex as well as orbitofrontal cortex (Kaufman et al.). Furthermore, Li and Zhao examined the aging effects on inter-region functional connectivity during bottom-up and top-down attention. In addition, Li et al. showed that the amplitude of P2 and P300 of MCI patients were weaker than normal subjects during retrieval period, and the P2 amplitude in retrieval period was positively correlated to the scores of memory test and visual spatial examination (Li et al.).

The second set of studies focus on the brain networks of MCI and Alzheimer's disease (AD) patients based on magnetic resonance imaging (MRI). A bunch of studies have examined the alterations of resting-state functional connectivity (rsFC) of MCI and/or AD patients from normal controls. However, limited reproducibility has been shown across studies which may have different study protocols. Tam et al. analyzed four different resting state functional MRI (rs-fMRI) data together, and reported reduced rsFC within areas of the default mode network and cortico-striatal-thalamic loop in amnestic MCI (aMCI) compared to normal controls. In another study, Kim et al. investigated the network properties of subjects including normal controls, aMCI patients, and prodromal and intermediate stages of AD patients with rs-fMRI data, and demonstrated that an unexpected stage-specifically non-monotonic reorganization of brain networks in the progression to AD. Wei et al. proposed a classification framework based on support vector machine to identify MCI converters (patients that covert to AD) from the MCI non-converters, and demonstrated that short-term prediction (6 and 12 months) resulted in better performance than long-term prediction.

The third set of studies are on the intervention effects to brain networks in aging. Jiang et al. investigated the cognitive training effects on cortical thickness and cognitive function for normal older subjects received single-domain or multi-domain cognitive training of 24 sessions. They observed significant interaction effect between group and time in the cortical thickness of the left supramarginal and the left frontal pole cortical regions, and old subjects received more benefits from multi-domain cognitive training than single-domain cognitive training (Jiang et al.). Cao et al. reported maintained or increased functional connectivity within the default mode network, salience network, and central executive network in older subjects after multi-domain cognitive training. Furthermore, Luo et al. observed significant cognitive training effects on lateralization of brain activities: the lateralization of the left frontoparietal network was retained in the training group but decreased in the control group. These studies accumulate new evidence for developing possible interventions to retain cognitive abilities in aging subjects.

Moreover, discussions on neuromodulation induced plasticity to in older adults (Shpektor et al., Puri and Hinder), MicroRNAs in pathologies of Alzheimer's disease (AD) (Ye et al.), and brief introduction on the applications of sparse representation-based classification in cognitive impairment and other brain diseases (Wen et al.) have been enrolled in this research topic. We wish these works would expand the technologies and methods for exploring brain networks in aging.

In summary, this Research Topic gathered a group of studies mainly on brain networks in normal older subject as well as MCI and AD patients. Studies demonstrated that normal older adults undertook decay in response inhabitation, attention control, and memory, which were accompanied by altered ERP components or functional connectivity compared with younger adults. For MCI or AD patients, alteration of their brain networks compared to normal older controls were observed, and the alterations may serve as potential biomarker in predicting the conversion of MCI to AD. In addition, the three studies in the third set investigated the effects of cognitive training to normal older adults and provided new evidence for developing interventions to retain cognitive function of older adults in aging. All these studies would enrich our understanding of neural mechanisms underlying aging.

## Author contributions

JS drafted the manuscript, and CL revised the manuscript.

### Conflict of interest statement

The authors declare that the research was conducted in the absence of any commercial or financial relationships that could be construed as a potential conflict of interest.

